# Transcriptome analysis of hen preadipocytes treated with an adipogenic cocktail (DMIOA) with or without 20(S)-hydroxylcholesterol

**DOI:** 10.1186/s12864-015-1231-z

**Published:** 2015-02-18

**Authors:** Alemu Regassa, Woo Kyun Kim

**Affiliations:** Department of Animal Science, University of Manitoba, Winnipeg, Manitoba Canada; Department of Poultry Science, University of Georgia, 303 Poultry Science Building, Athens, GA 30602 U.S.A

**Keywords:** Adipogenesis, Hen preadipocytes, 20(S)-hydroxycholestrol, Microarray

## Abstract

**Background:**

20(S)-hydroxycholesterol (20(S)) potentially reduces adipogenesis in mammalian cells. The role of this oxysterol and molecular mechanisms underlying the adipogenesis of preadipocytes from laying hens have not been investigated. This study was conducted to 1. Analyze genes differentially expressed between preadipocytes treated with an adipogenic cocktail (DMIOA) containing 500 nM dexamethasone, 0.5 mM 3-isobutyl-1-methylxanthine, 20 μg/mL insulin and 300 μM oleic acid (OA) and control cells and 2. Analyze genes differentially expressed between preadipocytes treated with DMIOA and those treated with DMIOA + 20(S) using Affymetrix GeneChip® Chicken Genome Arrays.

**Results:**

In experiment one, where we compared the gene expression profile of non-treated (control) cells with those treated with DMIOA, out of 1,221 differentially expressed genes, 755 were over-expressed in control cells, and 466 were over-expressed in cells treated with DMIOA. In experiment two, where we compared the gene expression profile of DMIOA treated cells with those treated with DMIOA+20(S), out of 212 differentially expressed genes, 90 were over-expressed in cells treated with DMIOA, and 122 were over-expressed in those treated with DMIOA+20(S).

Genes over-expressed in control cells compared to those treated with DMIOA include those involved in cell-to-cell signaling and interaction (IL6, CNN2, ITGB3), cellular assembly and organization (BMP6, IGF1, ACTB), and cell cycle (CD4, 9, 38). Genes over-expressed in DMIOA compared to control cells include those involved in cellular development (ADAM22, ADAMTS9, FIGF), lipid metabolism (FABP3, 4 and 5), and molecular transport (MAP3K8, PDK4, AGTR1). Genes over-expressed in cells treated with DMIOA compared with those treated with DMIOA+20(S) include those involved in lipid metabolism (ENPP2, DHCR7, DHCR24), molecular transport (FADS2, SLC6A2, CD36), and vitamin and mineral metabolism (BCMO1, AACS, AR). Genes over-expressed in cells treated with DMIOA+20(*S*) compared with those treated with DMIOA include those involved in cellular growth and proliferation (CD44, CDK6, IL1B), cellular development (ADORA2B, ATP6VOD2, TNFAIP3), and cell-to-cell signaling and interaction (VCAM1, SPON2, VLDLR).

**Conclusion:**

We identified important adipogenic regulators and key pathways that would help to understand the molecular mechanism of the in vitro adipogenesis in laying hens and demonstrated that 20(S) is capable of suppressing DMIOA-induced adipogenesis.

**Electronic supplementary material:**

The online version of this article (doi:10.1186/s12864-015-1231-z) contains supplementary material, which is available to authorized users.

## Background

Adipogenesis is the process in which preadipocytes become adipocytes, and it is one of the most intensively studied models of cellular differentiation. Adipocytes play vital roles in energy homeostasis and possess the largest energy reserve in the body of animals [1]. The increase in adipose tissue mass results from multiplication of fat cells through a process called adipogenesis, where undifferentiated precursor cells (preadipocytes) differentiate into fat cells [2].

A number of key transcriptional activities are involved in the process of adipogenesis in mammals [3-5]. The critical step in these events is the activation of the transcription factor CCATT enhancer-binding protein beta (C/EBPβ) by mitogen activated protein kinase (MAPK) and glycogen synthase kinase-3 beta (GSK3β) [6]. The activated C/EBPβ then triggers transcription of peroxisome proliferator-activated receptor gamma 2 (PPARγ2) and CCATT enhancer-binding protein alpha (C/EBPα), which in turn additively activate the expression of genes responsible for the development of mature adipocytes [3].

Oleic acid (OA) has been implicated as a good source of exogenous fatty acids essential for adipocyte differentiation and plays an important role in the development of adipose tissue in chickens [7]. Preadipocytes isolated from broilers (meat-type chicken) treated with 300 μM OA showed marked increase in the expression of genes responsible for adipocyte formation [7].

An adipogenic cocktail containing 500 nM dexamethasone, 0.5 mM 3-isobutyl-1-methylxanthine, and 20 μg/mL insulin (DMI) has been commonly used to induce adipogenesis in various animal models [3,4,8,9]. However, DMI treatment without OA does not induce key adipogenic transcription factors and adipogenesis in chicken preadipocytes [7,10] explaining the unique characteristic of chicken fat cell adipogenesis in vitro.

Oxysterols are bioactive molecules involved in numerous biological processes including cholesterol efflux [11], lipoprotein and calcium metabolisms [12], cell differentiation [13], and apoptosis [14] and are potential candidates for changing the fate of mesenchymal stem cell (MSC) differentiation [15]. While inhibiting adipogenic differentiation, specific oxysterols namely, 20(S)-hydroxycholesterol (20(S)) in combination with 22(S) - or 22(R)-hydroxycholesterol, induce osteoblastic differentiation of mouse pluripotent mesenchymal cells [16] through protein kinase C (PKC) and protein kinase A (PKA) dependent mechanisms [17]. These pro-osteogenic and anti-adipogenic effects of specific oxysterols are marked by the early and late markers of osteogenic differentiation such as increased alkaline phosphatase (ALP) activity, osteocalcin (OCN) mRNA expression and mineralization, and reduction in markers of adipogenic differentiation including lipoprotein lipase (LPL) and fatty acid binding protein 4 (FABP4) mRNA expression and adipocyte formation [16]. Furthermore, 20(S) inhibits PPARγ2 expression and adipogenic differentiation of mouse bone marrow stromal cells through a hedgehog (Hh)-dependent mechanism [15]. Similarly, treatment of mouse M2-10B4 MSC with Oxy34 or Oxy49 induces the expression of osteogenic differentiation markers, Runx2, Osterix (OSX), ALP, bone sialoprotein (BSP), and OCN as well as ALP enzymatic activity and robust mineralization [18]. On the other hand, treatment of these cells with the oxysterols inhibits the expression of adipogenic genes such as (PPARγ2), LPL, and FABP4, and adipocyte formation induced by PPARγ2 activator, troglitazone [18]. Additionally, treatment of human adipose-derived stem cells with 7-Ketocholesterol and 5,6-S oxysterols has been reported to detrimentally modulate mitochondrial activity and adipogenic differentiation of adipose precursor cells [19]. However, no studies have been conducted to elucidate global gene regulation of adipogenesis and anti-adipogenic mechanisms of oxysterols in chicken preadipocytes. Here, we hypothesized that treatment of hen preadipocytes with 20(S) reduces DMIOA-induced adipogenesis by affecting various pathways and gene networks involving key adipogeneic transcription factors. This study was conducted to identify genes differentially expressed between preadipocytes treated with DMIOA and control cells and genes differentially expressed between preadipocytes treated with DMIOA alone and those treated with a combination of DMIOA and 20(S).

## Methods

### Care of experimental birds

All experimental procedures were reviewed and approved by the University of Manitoba Animal Care Protocol Management and Review Committee, and birds were handled in accordance with guidelines described by the Canadian Council on Animal Care (CCAC, 1993).

### Cell culture

Abdominal adipose tissues weighing approximately 4 gm were collected from three 19-wk old laying hens (*Gallas gallus*, Lohman strain) by sterile dissection as described in [7]. We selected 19-wk old laying hens because hens start laying eggs, maximize their fat accumulation in the body and have dramatic metabolic changes related to lipid metabolism at this age. The adipose tissues were minced into fine sections with scissors and incubated in 10 mL Dulbecco’s Modified Eagle’s Medium (DMEM) digestion buffer containing 0.1% collagenase, 2.8 mM glucose, and 4% bovine serum albumin (BSA) for 45 min at 37°C in a shaking water bath. After the incubation, the contents were filtered using 100 and 40 μm Nylon meshes (Fisher Scientific, China), and the filtrates were centrifuged at 1,800 rpm for 10 min to separate floating adipocytes from pellets of preadipocytes. The supernatant was discarded, and cell pellets were resuspended in 10 mL 1X DMEM containing 10% fetal bovine serum (FBS), 100 U/mL penicillin, 100 μg/mL streptomycin and L-glutamate (Mediatech, Inc., Manassas, VA). Preadipocytes were then seeded in 100 mm Petri dishes (MG Scientific, Wisconsin, USA) and cultured in an incubator with 95% air and 5% CO_2_ at 37°C. Cells were checked for viability every day, and the media were changed every three days until the cells were confluent. At confluence, the cells were washed twice in 5 mL phosphate buffered saline (PBS) and incubated in 3 mL of 1X Tris-EDTA (TE) buffer for 2 min at 37°C. The cells were washed several times in seven mL of 1X DMEM containing 10% FBS to detach adhering cells. The contents were centrifuged at 1,800 rpm for 5 min, the supernatant was discarded, and cell pellets were re-suspended in 5 mL of 1X DMEM containing 10% FBS. Then, the cells were plated in six-well plates at 20,000 cells/cm^2^ and incubated until they reached confluence. The cells became confluent after two days of incubation.

### Incubation of cells with an adipogenic cocktail and 20(S)-hydroxycholesterol

**Figure 1:**
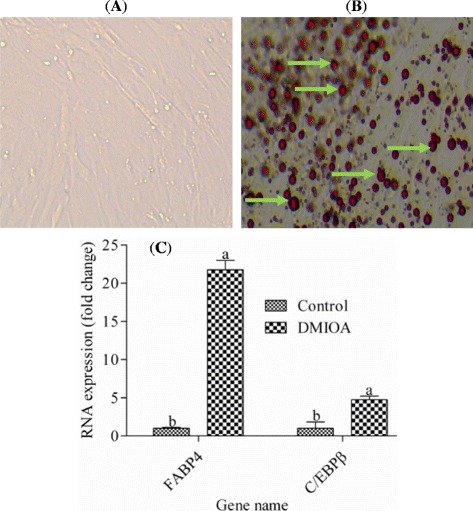
**Representative images of non-treated preadipocytes (1A), preadipocytes treated with DMIOA (1B), showing remarkable lipid accumulation in preadipocytes treated with DMIOA and relative expressions of FABP4 and C/EBPB in control and DMIOA treated cells (1C).** Preadipocytes were cultured in Dulbecco’s modified eagle’s medium (DMEM) containing 10% fetal bovine serum (FBS) for 96 hr. Images were taken using an EVOS® xl core cell culture microscope (Advanced Microscopy Group, Seattle, USA) at 20X magnification. Green arrows indicate lipid droplets stained with Oil red O stain whereas there is no lipid formation in non-treated cells. **C:** Fold change expression of FABP4 and C/EBPβ in preadipocytes treated with an adipogenic cocktail (DMIOA) compared with non-treated cells. Preadipocytes were cultured in Dulbecco’s modified eagle’s medium containing 10% Fetal Bovine Serum for 96 hr. Bars with different letters are significantly different (P < 0.05). The bars represent Mean ± SD (N = 3).

**Figure 2:**
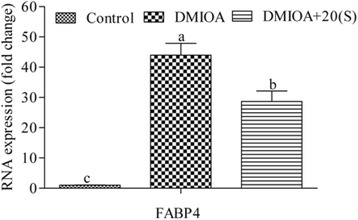
**Relative expression of FABP4 in non-treated (control) preadipocytes, preadipocytes treated with an adipogenic cocktail (DMIOA) and those treated with DMIOA + 20(S)-hydroxycholestrol in Dulbecco’s modified eagle’s medium containing 10% Fetal Bovine Serum for 96 hr.** Bars with different letters are significantly different (P < 0.05). The bars represent Mean ± SD (N = 3).

After confluence, the cells were treated with 1) an adipogenic cocktail containing 500 nM dexamethasone, 0.5 mM 3-isobutyl-1-methylxanthine, 20 μg/mL insulin and 300 μM OA (DMIOA), and 2) DMIOA + 5 μM 20(S) with three biological replicates per treatment for 96 hr. Cells were incubated for 96 hr because our recent observation showed that sufficient lipid droplets were formed by DMIOA treatment and 20(S) started inhibiting adipogenic gene (FABP4) at this time point (Figures 1 and 2). Non-treated cells were cultured in 1X DMEM containing 10% FBS without an adipogenic cocktail for comparison purpose. The media was removed from the cells using a vacuum aspirator, and the cells were homogenized in one mL of TRIzol (Invitrogen, Canada) for RNA isolation.

### Oil red O staining of preadipocytes

In order to examine the effect of an adipogenic cocktail on adipogenesis and accumulation of lipid droplets, preadipocytes isolated from 19-wk old laying hens were treated with DMIOA containing 500 nM dexamethasone, 0.5 mM 3-isobutyl-1-methylxanthine, 20 μg/mL insulin and 300 μM OA for 96 hr. Then, non-treated control cells and cells treated with DMIOA were stained with Oil Red O according to supplier’s protocol [20].

### RNA isolation and array processing

Total RNA was extracted from cells representing three biological replicates per treatment using TRIzol (Invitrogen, Canada) according to the manufacturer’s protocol. RNA integrity and yield of each sample were determined using an Experion™ automated electrophoresis system (Bio-Rad laboratories Inc, USA) and a NanoDrop 2000 (Thermo Scientific, Canada), respectively. Initial total RNA concentrations across all samples were adjusted to 500 ng, and this amount of total RNA was used for the two round cDNA synthesis and subsequent in vitro-transcription according to the one-cycle eukaryotic target labeling assay (Affymetrix GeneChip® 3′ IVT express Kit). Fifteen μg of biotin-labeled and amplified RNA (aRNA) of each group were hybridized with Affymetrix GeneChip® Chicken Genome Arrays for 16 hr at 45°C. Post-hybridization staining and washing were performed according to manufacturer’s protocols using a Fluidics Station 450 instrument (Affymetrix, USA).

### Image capturing, quantification and data analysis

Array slides were scanned with a GeneChip™ 3000 laser confocal slide scanner (Affymetrix, USA), and the images were quantified using Affymetrix GeneChip Command Console Software (Affymetrix, USA). Probe level data were imported into FlexArray software [21]. The raw data were background corrected, normalized, and summarized using Guanine-Cytosine Robust Multichip Average (GCRMA) function as described in [22]. The background corrected and normalized data were then filtered by applying a false discovery rate of < 5%. The filtered genes were annotated using a chicken annotation file (Chicken. na32.annot, Affymetrix, USA). Genes differentially expressed (1) between non-treated (control) cells and cells treated with DMIOA and (2) between cells treated with DMIOA alone and a combination of DMIOA and 20(S) were identified using a t-test at a fold change of ≥ 2 and probability (P < 0.05). The raw data from all arrays in this study are available online at http://www.ncbi.nlm.nih.gov/geo/ with GEO accession number GSE50880.

Lists of genes over-expressed in either group were uploaded into Ingenuity Pathways Analysis [23] to identify the most significantly affected gene networks and cellular functions, relationships between the genes of interest, and pathways involved.

### Validation of microarray data using quantitative real-time reverse transcription polymerase chain reaction (qRT-PCR)

The same RNA samples that were used for array hybridization were used for cDNA synthesis. First strand cDNA was synthesized using a high capacity reverse transcription kit according to the supplier’s protocol (Applied Biosystems, Canada). Pairs of primers for each gene were designed from the mRNA sequence of target gene using the National Centre for Biotechnology Information (NCBI). Quantitative real-time RT-PCR was performed in duplicate reactions including nuclease free water, the forward and reverse primers of each gene, template cDNA and SYBR Green using a CFX Connect ™ Real-Time PCR Detection System (Life Science Research, Bio-Rad, Canada). Data were generated using a ∆∆Ct method by normalizing the expression of the target genes to a housekeeping gene (GAPDH), and the values were reported as fold changes of the expression of the target genes in DMIOA treated cells compared with the control group and the expression of target genes in DMIOA + 20(S) treated cells compared with DMIOA group. Gene expression and correlation between microarray and qRT-PCR data were analysed using t-test and correlation procedures of SAS software [24], respectively. Means were declared significant at P < 0.05. Pairs of primers used for qRT-PCR assay and their sequences are presented in Table 1.

**Table 1:** **List of primers used for quantitative real-time polymerase chain reaction**

**Name**	**Forward**	**Reverse**	**Product length (base pair)**	**Annealing temperature (°C)**
FABP4	GAGTTTGATGAGACCACAGCAGA	ATAACAGTCTCTTTGCCATCCCA	106	57
IGFBP7	TCCATCGTGACCCCTCCTAA	GAGAGATCAGTACCCAGCCG	228	55
VEGFc	AAGTGTGTGTGGATGTGGGG	TGACAGTTACGGGTTTGGGG	201	55
CD44	CGCTGTGCGGAGATACAGAA	CCTATGGCTCTTCCTGGCTG	199	55
MMP1	GCAGTCTCCTCTGCTTTCCC	GTCACGGTCAGGTTTCCCAG	209	58
STARD4	GGGACAGCACAAGCCCTAAT	GCCTAGCTTGACTGGGTTCA	219	54
INSIG1	GCATGGTGCCAGTGTGAAAG	TCCAGAGAACAGCCATACGC	222	54
GAPDH	GCTAAGGCTGTGGGGAAAGT	TCAGCAGCAGCCTTCACTAC	116	55
TGM4	TGGATGTCCTCTGACTCCGT	CAGTAGACCTTGTCGGCGTT	238	54
CCL4	CTCATGGCAGGTGCTGTTTG	CCTCCCTTAAATGCCCTCCC	207	55
TNFAIP3	CAGAAAAGAGGCCTGCTCCA	CCTTCAGTTTCTCGGGTGCT	202	54
GSTA3	GCCAAAGGAAACCACGCCTA	GTTTCATCCAGTGTACCGCCT	218	55
APOA1	CTCGCTGTGCTCTTCCTGAC	GTCAGCCAGCTTCAGGTCAA	191	55
KLF2	CTTACCCGCCACTACCGAAA	TTGTCCGGCTCTGTCCTAAG	123	58
HAS2	CACTGGGAGAAGCGTGGAAT	GCACTGTACGCAGCCAAAAT	203	56

## Results

### The effect of DMIOA on the adipogenesis and expression of key adipogenic transcripts

The results of our pre-experiment investigation indicated that, preadipocytes treated with DMIOA had remarkably higher lipid accumulation (Figure 1B) and significantly higher (P < 0.05) expression of key adipogenic transcripts such as FABP4 and C/EBPβ compared with non-treated cells (Figure 1C).

### The effect of 20(S)-hydroxycholesterol (20(S)) on the expression of FABP4

In order to study the effect of oxysterol on the expression of adipogenic transcripts, preadipocytes isolated from 19-wk laying hens were treated with DMIOA with or without 20(S). The result indicated that DMIOA treatment significantly increased (P ≤ 0.05) the expression of one of the key adipogenic transcript (FABP4) compared to control cells, whereas DMIOA + 20(S) treatment significantly reduced DMIOA-induced FABP4 expression (Figure 2).

### Transcriptome profiles of chicken preadipocytes treated with DMIOA relative to non-treated (control) cells

Global transcriptome expression analyses of preadipocytes treated with DMIOA and non-treated cells showed that of 1,221 differentially expressed genes, 755 were over-expressed in non-treated cells (Additional file 1), and 466 were over-expressed in cells treated with DMIOA (Additional file 2). Hierarchical clustering of all genes differentially expressed between control cells and cells treated with DMIOA and the top 20 genes differentially expressed between the two groups are indicated in Additional files 3 and 4, respectively.

**Figure 3:**
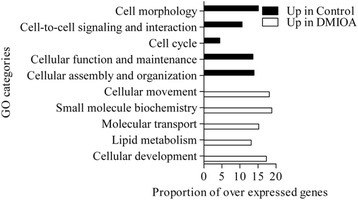
**The top significantly changed gene ontology (GO) terms (molecular and cellular functions) with the proportion of genes involved among these over-expressed in non-treated preadipocytes (black bars) and those treated with an adipogenic cocktail (DMIOA) (white bars) in Dulbecco’s modified eagle’s medium containing 10% Fetal Bovine Serum for 96 hr.** Lists of genes over-expressed in a given treatment group relative to the other were imported into IPA and the number of genes that were associated with a given GO term in the IPA database was determined at P < 0.0001. The P-value, calculated using the right-tailed Fisher Exact Test, is a measure of the likelihood that the association between the number of focus genes in the data set and a given GO term is due to random chance. The smaller the P-value is the less likely that the association is random, and the more significant the association is. The p-value for a given GO term was calculated by considering the number of focus genes that participate in a given GO term and the total number of genes that are known to be associated with that GO term in the Ingenuity Knowledge Base. Then, the proportion of genes in a given GO term was calculated by dividing the number of genes determined by IPA that are associated with a given GO term in the Ingenuity® Knowledge Base by the total number of genes over-expressed in one treatment group relative to the other multiplied by 100.

Genes over-expressed in control cells compared to those treated with DMIOA include those involved in cell-to-cell signaling and interaction such as interleukin 6 (IL6), calponin 2 (CNN2), and integrin beta 3 (ITGB3), cell morphology such as ATPase, Ca^++^ transporting, plasma membrane 2 (ATP2B2), insulin like growth factor 3 (IGFB3), and inhibin beta A (INHBA), cellular assembly and organization such as bone morphogenetic protein 6 (BMP6), insulin like growth factor 1 (IGF1), beta actin (ACTB), and adenylyl cyclase-associated Protein 2 (CAP2), cellular function and maintenance such as integrin alpha 4 (ITGA4), integrin beta 2 (ITGB2), and growth and differentiation factor 9 (GDF9), and cell cycle such as CD4, 9 and 38 molecules (CD4, CD9, CD38), and cyclin-dependent kinase inhibitor 2B (CDKN2B). Genes over-expressed in DMIOA compared to control cells include those involved in cellular development such as ADAM metallopeptidase domain 22 (ADAM22), ADAM metallopeptidase with thrombospondin type 1 motif, 9 (ADAMTS9), c-fos induced growth factor (FIGF), and matrix metallopeptidase 1 (MMP1), lipid metabolism such as fatty acid binding protein 3, 4, and 5 (FABP3, 4 and 5), and apolipoprotein 1 (APOA1), molecular transport such as mitogene activated protein 3 kinase 8 (MAP3K8), pyruvate dehydrogenase kinase, isozyme 4 (PDK4), solute carrier organic anion transporter family, member 2B1 (SLCO2B1), and nicotinamide phosphoribosyltransferase (NAMPT), and small molecule biochemistry such as angiotensin II receptor, type 1, 2 and 3 (ATGR1, ATGR2, ATGR3), and glutamine-fructose-6-phosphate transaminase 2 (GFPT2) (Figure 3).

**Figure 4:**
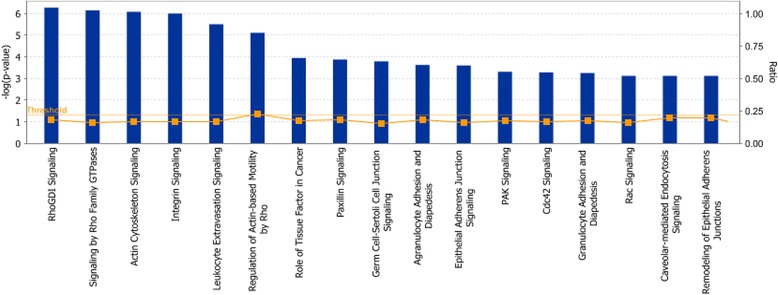
**Functional grouping of genes that were over-expressed in non-treated preadipocytes relative to those treated with an adipogenic cocktail (DMIOA) showing the most significant functional groups, with P value ≤ 0.05.** The bars represent the P-value in a logarithmic scale for each functional group.

**Figure 5:**
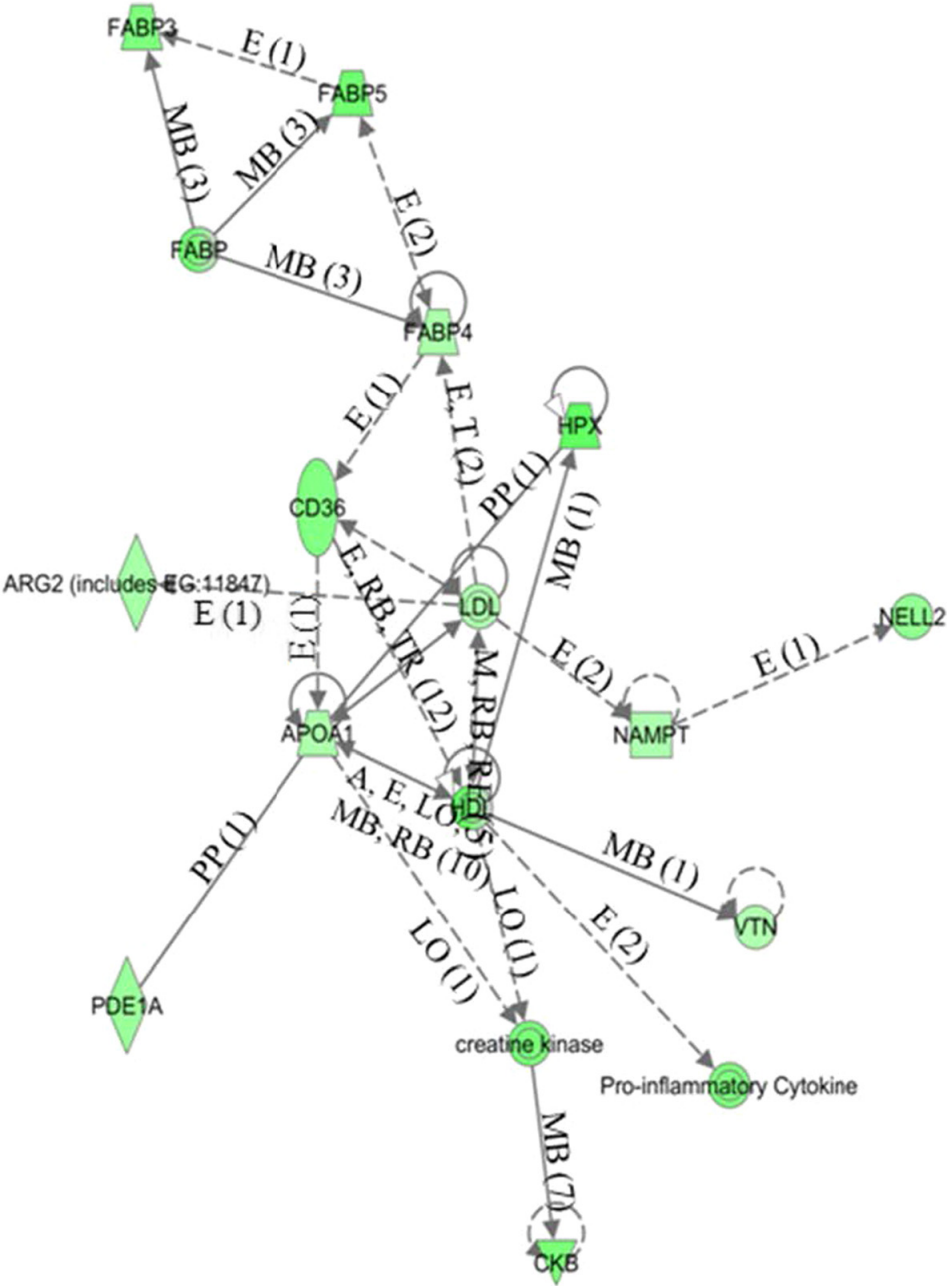
**An example of a gene network (lipid metabolism) showing the relationships between molecules over-expressed in cells treated with an adipogenic cocktail (DMIOA) compared with non-treated control cells.** The type of the association between two molecules is shown by a letter on the line that connects them. The number in parenthesis next to the letter represents the number of bibliographic references currently available in the Ingenuity Pathways Knowledge Base that support each one of the relationships. Direct or indirect relationships between molecules are indicated by solid or dashed lines connecting them, respectively. P = phosphorylation, A = gene activation, E = increase in expression, PP = protein-protein interaction, MB = membership in complex, LO = localization, L = proteolysis, RB = regulation of binding, TR = Translocation and T = Transcription. Rhombus, triangular, rectangular, oval, and circular shapes indicate that the molecule belongs to a family of enzymes, phosphatases, growth factors, transmembrane receptors, and other families, respectively.

Functional grouping (canonical pathways) of genes that were over-expressed in control preadipocytes relative to those treated with DMIOA is presented in Figure 4. Several key gene networks were also affected by DMIOA treatment relative to control cells. An example of a gene network (lipid metabolism) showing the relationships between molecules over-expressed in preadipocytes treated with DMIOA compared with control cells is presented Figure 5.

### Transcriptome profile of chicken preadipocytes treated with DMIOA alone and a combination of DMIOA and 20(S)

The gene expression data indicated that of 212 differentially expressed genes, 90 were over-expressed in cells treated with DMIOA alone (Additional file 5), and 122 were over-expressed in those treated with a combination of DMIOA and 20(S) (Additional file 6). Hierarchical clustering of all genes differentially expressed between cells treated with DMIOA and those treated with DMIOA + 20(S) and the top 20 genes differentially expressed between the two groups are indicated in Additional files 7 and 8, respectively.

**Figure 6:**
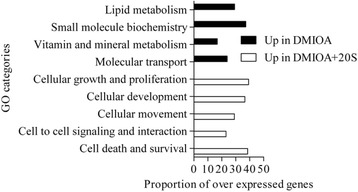
**The top significantly changed GO terms (molecular and cellular functions) with the proportion of genes involved among these over-expressed in cells treated with an adipogenic cocktail (DMIOA) (black bars) and those treated with a combination of DMIOA and 5 μM 20(S) (white bars) relative to each other.** Lists of genes over-expressed in a given treatment group relative to the other were imported into IPA and the number of genes that were associated with a given GO term in the IPA data base was determined at P ≤ 0.0001. The P-value, calculated using the right-tailed Fisher Exact Test, is a measure of the likelihood that the association between the number of focus genes in the data set and a given GO term is due to random chance. The smaller the P-value is the less likely that the association is random and the more significant the association is. The p-value for a given GO term was calculated by considering the number of focus genes that participate in a given GO term and the total number of genes that are known to be associated with that GO term in the Ingenuity Knowledge Base. Then, the proportion of genes in a given GO term was calculated by dividing the number of genes determined by IPA that are associated with a given GO term in the Ingenuity® Knowledge Base by the total number of genes over-expressed in one treatment group relative to the other multiplied by 100.

**Figure 7:**
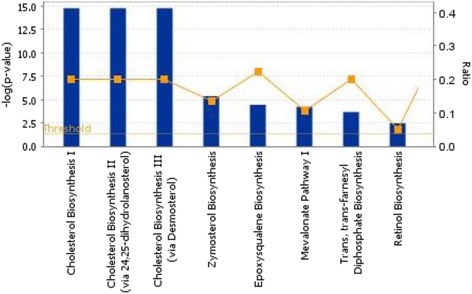
**Functional grouping of genes that were over-expressed in cells treated with an adipogenic cocktail (DMIOA) relative to those treated with a DMIOA + 5 μM 20(S)-hydroxycholesterol showing the most significant functional groups, with P values, 0.05.** The bars represent the P-value in a logarithmic scale for each functional group.

**Figure 8:**
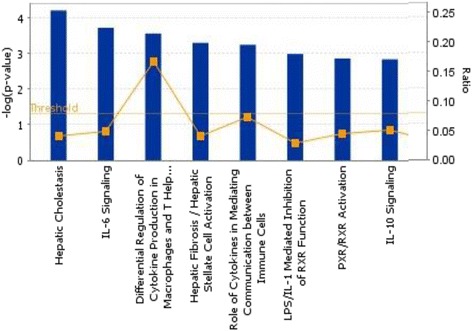
**Functional grouping of genes that were over-expressed in preadipocytes treated with an adipogenic cocktail (DMIOA) + 5 μM 20(S)-hydroxycholesterol relative to those treated with DMIOA alone showing the most significant functional groups, with P values, 0.05.** The bars represent the P-value in a logarithmic scale for each functional group.

Genes over-expressed in cells treated with DMIOA alone compared with those treated with DMIOA + 20(S) include those involved in lipid metabolism such as ectonucleotide pyrophosphatase/phosphodiesterase 2 (ENPP2), 7-dehydrocholesterol reductase (DHCR7), 24-dehydrocholesterol reductase (DHCR24), 3-hydroxy-3-methylglutaryl-CoA reductase (HMGCR), farnesyl-diphosphate farnesyltransferase 1 (FDFT1), and farnesyl diphosphate synthase (FDPS), small molecule biochemistry such as solute carrier family 16 member 10 (SLC16A10), fibroblast growth factor 7 (FGF7), StAR-related lipid transfer (START) domain containing 4 (STARD4), and insulin induced gene 1 (INSIG1), molecular transport such as fatty acid desaturase 2 (FADS2), solute carrier family 6 member 2 (SLC6A2), glypican 1 (GPC1), and CD molecule 36 (CD36), and vitamin and mineral metabolism such as beta-carotene 15,15′-monooxygenase (BCMO1), acetoacetyl-CoA synthetase (AACS), androgen receptor (AR), and hydroxysteroid (17-beta) dehydrogenase 7 (HSD17B7). Genes over-expressed in cells treated with DMIOA + 20(S) compared with those treated with DMIOA alone include those involved in cellular growth and proliferation such as CD molecule 44 (CD44), cyclin dependent kinase 6 (CDK6), interleukin 1 beta (IL1B), interleukin 6, and 8 (IL6, and 8), cellular development such as adenosine A2b receptor (ADORA2B), ATP synthase (ATP6), tumor necrosis alpha induced protein 3 (TNFAIP3), and tumor necrosis factor (ligand) superfamily, member 15 (TNFSF15), cellular movement such as chemokine (C-C motif) ligand 20 (CCL20), cholecystokinin (CCK), and vasoactive intestinal polypeptid (VIP), and cell-to-cell signaling and interaction such as vascular cell adhesion molecule 1 (VCAM1), spondin 2, extracellular matrix protein (SPON2), very low density lipoprotein receptor (VLDLR), and cytoplasmic polyadenylation element binding protein 1 (CPEB1). The gene ontology (GO) terms (cellular and molecular functions) and functional groups (canonical pathways) of genes differentially expressed between cells treated with DMIOA alone and those treated with DMIOA + 20(S) are shown in Figures 6, 7 and 8, respectively. The results of functional (pathway) analysis indicated that the majority of genes over-expressed in cells treated with DMIOA relative to those treated with DMIOA + 20(S) are involved in cholesterol biosynthesis I, II and III pathways (Figure 7). On the other hand, the majority of genes over-expressed in cells treated with DMIOA + 20(S) are involved in hepatic cholestasis, IL-6, IL-10, and LPS (IL-1) mediated inhibition of RXR function (Figure 8).

**Figure 9:**
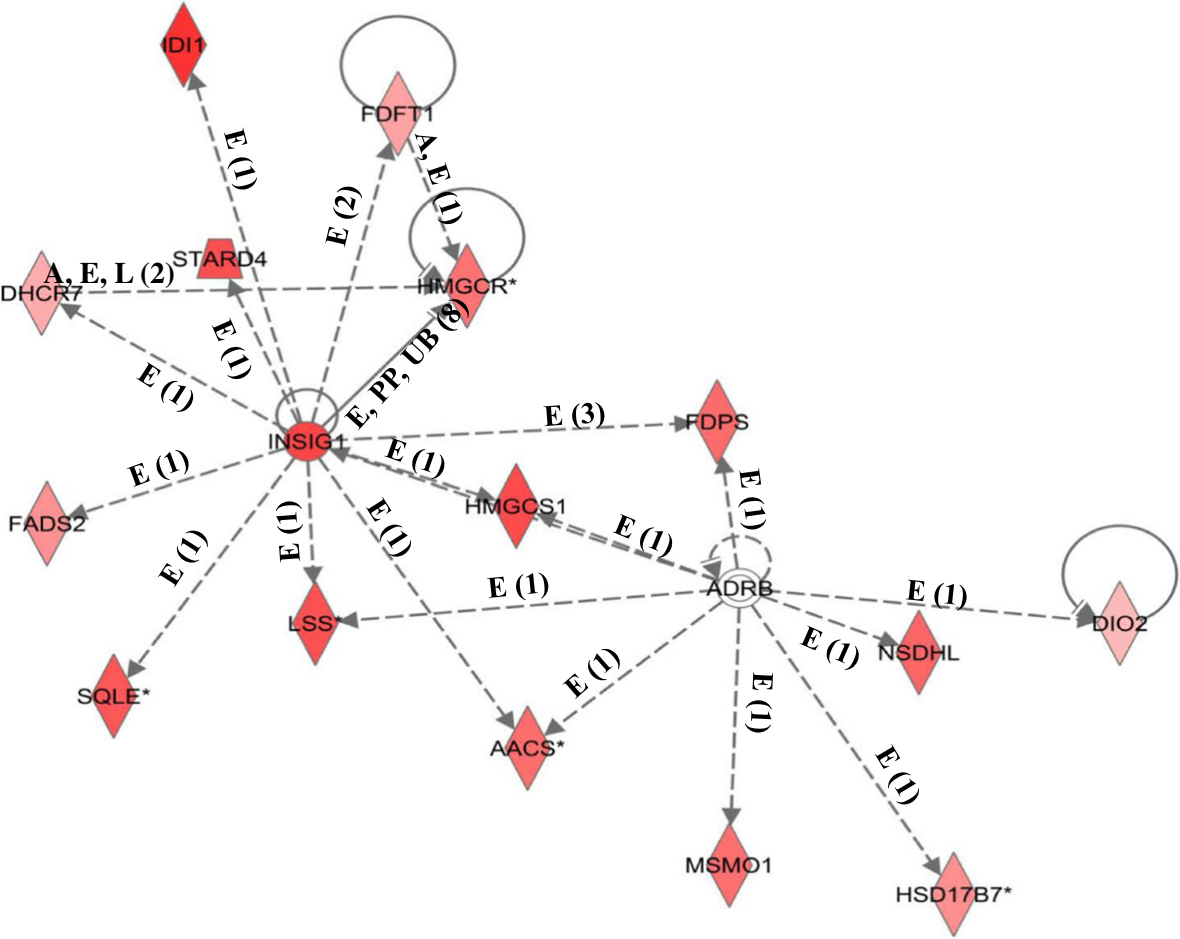
**An example of a gene network (lipid metabolism) showing the relationships between molecules over-expressed in cells treated with an adipogenic cocktail (DMIOA) compared with those treated with a combination of DMIOA and 5 μM 20(S)-hydroxycholesterol.** The type of the association between two molecules is shown as a letter on the line that connects them. The number in parenthesis next to the letter represents the number of bibliographic references currently available in the Ingenuity Pathways Knowledge Base that support each one of the relationships. Direct or indirect relationships between molecules are indicated by solid or dashed lines connecting them, respectively. A = activation, E = increase in expression, PP = protein-protein interaction, L = proteolysis, and UB = ubiquitination. Rhombus shape indicates that the molecule belongs to the family of enzymes, whereas circular shape indicates the molecule belongs to other families.

**Figure 10:**
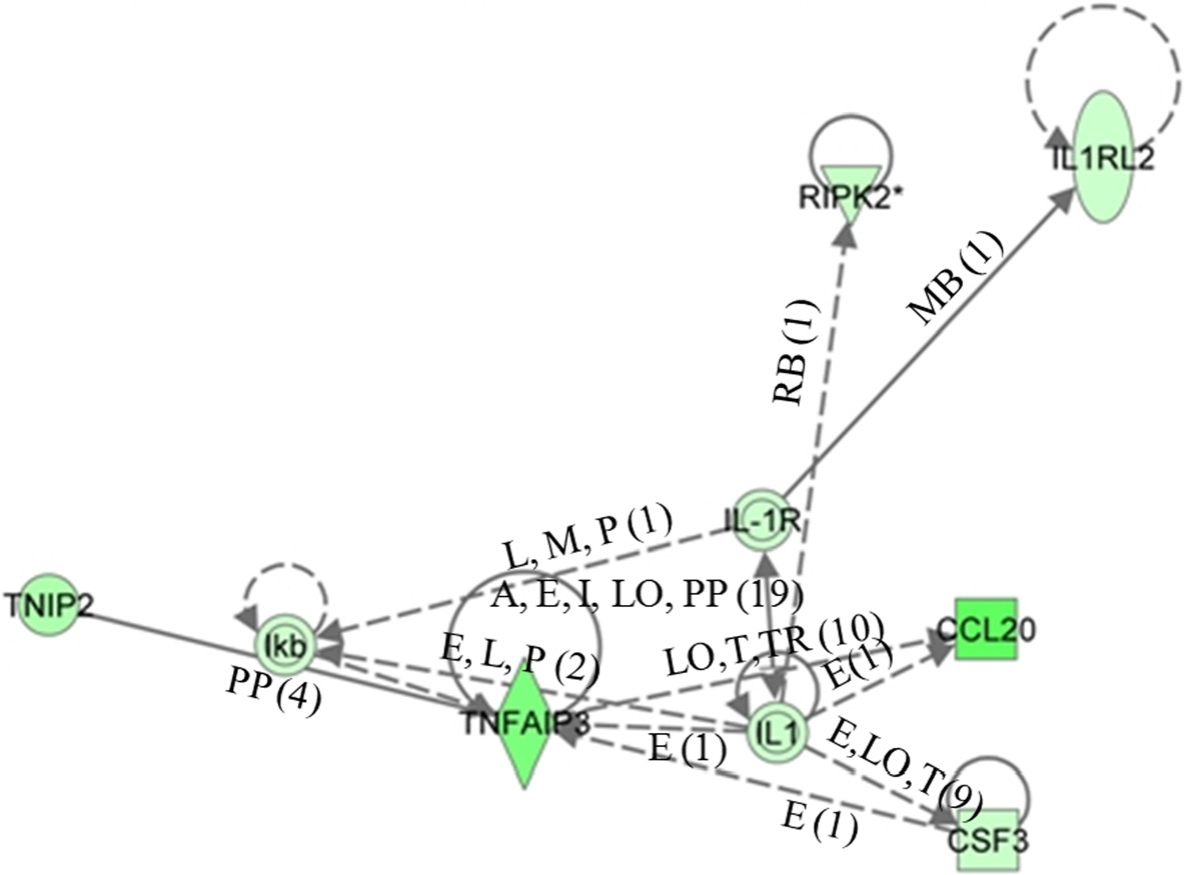
**An example of a gene network (cell-to-cell signaling) showing the relationships between molecules over-expressed in cells treated with an adipogenic cocktail (DMIOA) + 5 μM 20(S)-hydroxycholesterol compared with those treated with DMIOA alone.** The type of the association between two molecules is shown as a letter on the line that connects them. The number in parenthesis next to the letter represents the number of bibliographic references currently available in the Ingenuity Pathways Knowledge Base that support each one of the relationships. Direct or indirect relationships between molecules are indicated by solid or dashed lines connecting them, respectively. P = phosphorylation, A = gene activation, E = increase in expression, PP = protein-protein interaction, MB = membership in complex, LO = localization, L = proteolysis, RB = regulation of binding, TR = Translocation and T = Transcription. Triangular, rectangular, oval, and circular shapes indicate that, the molecule represented belongs to the family of phosphatases, growth factors, transmembrane receptors, and others, respectively.

Key gene networks showing the relationships between molecules over-expressed in cells treated with DMIOA and those treated with DMIOA + 20(S) relative to each other are presented in Figures 9 and 10. One of the gene networks that were affected by DMIOA treatment is a lipid metabolism gene network including signaling molecules such as INSIG1, STARD4, SQLE and others (Figure 9), whereas the network affected by DMIOA + 20(S) includes genes that are involved in interleukin signaling pathways such as IL-1, IL-1R, IL1RL2, TNFAIP3, CCL20 (Figure 10).

### Validation of microarray data using quantitative real-time reverse transcription polymerase chain reaction (qRT-PCR)

In order to validate the microarray data, we selected representative genes and analyzed their expression using qRT-PCR. Except for one gene, HAS2, the results were consistent with the microarray data. Additionally, correlation analysis of the microarray and qRT-PCR data showed a strong positive correlation (r = 0.989; P < 0.001) between the two data sets. Microarray and RT-PCR fold differences are shown in Table 2.

**Table 2:** **Fold change and probability values of qRT-PCR and micro array data**

**Gene name**	**Fold change**		**P value**	
	**RT-PCR**	**Micro array**	**RT-PCR**	**Micro array**
CD44	8.2	5.5	P = 0.02	P = 0.011
IGFBP7	12.1	9.1	P = 0.0002	P = 0.046
VEGFc	4.1	8.5	P = 0.0001	P = 0.027
MMP1	367	598.0	P = 0.001	P = 0.001
APOA1	61.0	12.2	P = 0.0009	P = 0.021
STARD4	4.0	6.2	P = 0.0008	P = 0.043
INSIG1	6.8	6.9	P = 0.0005	P = 0.012
TGM4	4.5	4.2	P =0.0004	P = 0.032
TNFAIP3	4.6	9.7	P = 0.001	P = 0.008
GSTA3	1.4	5.9	P = 0.04	P = 0.049
KLF2	6.5	3.9	P = 0.001	P = 0.04
HAS2	0.8	6.2	P = 0.15	P = 0.00048

CD44, IGFBP7, and VEGFc were genes over expressed in control cells and MMP1 and APOA1 were genes over expressed in cells treated with DMIOA relative to each other. Similarly, STARD4, INSIG1, and TGM4 were genes over expressed in DMIOA treated cells whereas TNFAIP3, GSTA3, and KLF2 were genes over expressed in cells treated with DMIOA + 20(S) relative to each other.

## Discussion

### The role of microarray in chicken transcriptomics

The use of microarray in chicken transcriptome analysis is recently increasing. For instance, microarray-based gene expression studies were conducted on adipose tissues collected from 7- [25] and 9- [26] wk old broiler chickens to identify genes differentially expressed between fat and lean lines. Systematic identification of candidate genes and new pathways related to intramuscular fat deposition in chicken breast tissues has also been made using gene expression profiles of two distinct breeds [27] showing the wide use of microarray in gene expression studies and identification of important pathways and biological processes in chicken.

### The effect of an adipogenic cocktail (DMIOA) on adipogenesis and expression of adipogenic genes

In the present study, we detected numerous genes over-expressed in preadipocytes treated with DMIOA compared with non-treated cells including ENPP2, RLN3, ADAMTS9, HPGD, LCN8, HPX, OLFM1, ST6GAL1, CHRDL2, CIDEC, CKB, AVPR2 and others (Additional file 2). Although some of the genes over-expressed in preadipocytes treated with DMIOA relative to non-treated cells are known to have roles in adipogenesis, the majority of them are genes with currently unknown function related to adipogenesis. Although we believe that the study of these genes may provide new insight about adipogenesis, only genes that are known to have direct or indirect roles in adipogenesis and involved in known pathways and molecular functions are discussed here.

Fatty acids have been implicated as potent inducers of adipogenic genes in mammalian [28,29] and chicken [7,10] preadipocytes. Fatty acids, certain prostaglandins, and prostaglandin metabolites are known to function as ligands of PPARγ2 to induce adipogenic differentiation [30]. Treatment of 3 T3-L1 mouse preadipocytes with medium size fatty acids increases the expression of adipocyte specific transcription factors, such as PPARγ2, C/EBPα and sterol regulatory element binding protein 1 C (SREBP1C), and the major adipocyte marker genes, such as FABP4 and glycerol-3-phosphate dehydrogenase (GPDH) [28]. Rapid increase in PPARγ2 and FABP4 mRNA expression has been reported in preadipocytes isolated from broiler chicken between 9 and 12 hr of OA treatment [7]. In the present study, PPARγ2 and C/EBPα mRNAs were not differentially expressed between cells treated with DMIOA and non-treated cells after 96 hr of incubation. These data are consistent with the findings of [7] where higher levels of PPARγ2 and C/EBPα mRNAs measured between 9–12 hr, and after 24 hr of incubation, respectively, were followed by a sharp decline, suggesting the induction of these genes during the early stages of differentiation as they were not differentially expressed in cells treated with DMIOA after 96 hr incubation in the present study.

Histone deacetylase 9 (HDAC9) was over-expressed in non-treated cells compared with those treated with DMIOA (Additional file 1). Similarly, over-expression of HDAC9 reduces C/EBPα expression and adipogenesis, whereas its knock-out enhances the expression of C/EBPα and accelerated adipogenesis in 3 T3-L1 cell lines, demonstrating the anti-adipogenic effect of this gene [31].

Higher expression of one of the key adipocyte specific transcription factors, C/EBPβ, was also measured in cells treated with DMIOA compared with non-treated control cells, and this is consistent with the findings of [7] where its expression level has been progressively increased after 24 hr of incubation, showing an increasing trend with incubation time. However, our data are not fully consistent with the results from 3 T3-L1 mouse preadipocytes study where higher expression of SREBP1C and GPDH activities have been reported [28], explaining the possible inter-species and incubation time differences in the characteristics of adipocyte differentiation.

The ingenuity pathway analysis indicated that treatment of preadipocytes with DMIOA increased the expression of genes involved in lipid metabolism, including CD36, AGTR2, MAP3K8, APOA1, PTX3, HMOX1 and others (Figure 3). CD36 is a fatty acid transporter molecule that has been implicated to play a functional role in the differentiation of 3 T3-F442A murine preadipocytes into matured adipocytes in in-vitro and in-vivo environments [32], and impaired fatty acid influx and triglyceride synthesis were reported in adipocytes lacking CD36 [33]. Higher expression of CD36 in cells treated with DMIOA compared with non-treated cells in the present study suggests the importance of this transcript in the adipogenesis of hen fat cells.

MAP3K8 is specifically involved in IL-1β and tumor necrosis alpha (TNF-α) activated MAPK pathway in adipocytes and up-regulated in adipose tissue of obese subjects [34]. Similarly, pentraxin 3 (PTX3) mRNA levels were higher in adipose tissue of genetically obese mice versus control mice [35]. However, whether this higher expression of PTX3 and MAP3K8 in adipose tissue of genetically obese subjects and in DMIOA treated cells is associated with their roles in adipogenesis is not well understood.

Another interesting gene highly expressed in DMIOA treated cells, but not in agreement with the result from mouse study, was heme oxygenase 1 (HMOX1). HMOX1 has been reported to lower the elevated levels of key adipogenic genes, such as PPARγ, FABP4, C/EBPβ, and Wnt5B, but increased the expression of anti-adipogenic transcription factors, such as sonic hedgehog (Shh), Wnt10B and β-catenin, in adipocytes isolated from mice fed a high fat diet [36]. However, whether this observed difference is attributed to inter-species differences or high fat diet need to be elucidated.

We found higher transcript expression for arginase 2 (ARG2), hemopexin (HPX), and vitronectin (VTN) in cells treated with DMIOA compared with non-treated cells. These genes are involved in liver X receptor (LXR)/Retinoid X receptor (RXR) activation pathway, indicating OA possibly induce adipogenesis in hen fat cell through activation of LXR/RXR pathways. The role/s of LXR/RXR pathway appears to vary from cells to cells and from species to species. For instance, suppression of LXRα markedly reduces the expression of lipogenic (FAS and SREBP1C) and adipogenic transcription factors (PPARγ2 and FABP4) and adipogenesis in 3 T3-L1 mouse cells [37]. Similarly, suppression of LXR negatively regulates adipogenesis in C57BL/6 mouse cells [38] suggesting that LXRα can be an important transcription factor mediating adipocyte differentiation as well as adipogenic gene expression. On the contrary, knock-down of LXRα and LXRβ in mouse M2 bone marrow stromal cells significantly reduced the expression of key osteogenic genes, BSP, OCN, and HES1, showing the critical role of LXRs in regulating mouse MSC osteogenic differentiation [39] that has a reciprocal interaction with adipogenic differentiation. In another study, activation of LXRα resulted in lipolysis of human 3 T3-L1 cells [40] showing inter-species differences.

We also found higher expression of genes involved in IL-10 signaling, such as IL-1R1, IL-1RL1, and IL-1RL2, and these involved in lipopolysaccharide (LPS)/IL-1 mediated inhibition of RXR including FABP3, 4, 5, and SOD3 pathways in cells treated with DMIOA compared with non-treated cells. Interleukin 10 (IL-10) is a potent anti-inflammatory and immunosuppressive cytokine mediating its pleiotropic effects on different immune cells through the trans-membrane heterotetrameric complex composed of IL-10R1 and IL-10R2 chains [41]. Adipose tissue is a rich source of anti-inflammatory factors such as IL-10 [42,43], and adipose size is an important determinant of adipokine secretion [44]. However, data showing the role of IL-10 signaling pathway itself in adipogenesis is not available and hence molecular mechanisms of the IL-10 and other signaling pathways in adipogenesis by DMIOA treatment is to be elucidated.

Fatty acid binding proteins (FABP3, 4 and 5) play important roles in adipogenic differentiation of fat cells in various species [7,10,45]. Consistent with this, DMIOA treatment increased the expression of molecules involved in the lipid metabolism gene network such FABP3, 4 and 5, APOA1, LDL, and others (Figure 5). Although both FABP4 and FABP5 are expressed in adipose tissue, only the regulatory role of FABP4 in adipogenesis has been widely investigated. Increased expression of FABP4 has been reported in DMIOA-induced adipogenic differentiation of preadipocytes isolated from chicken [7,10]. A coordinated expression of FABP3, FABP4 and FABP5 together with that of PPARα, PPARγ1 and PPARγ2 is critical for metabolic regulation of adipogenesis in porcine preadipocytes [45]. Contrastingly, FABPs are not differentially expressed between fat and lean lines of 7-wk old broiler chicken [25].

It has been reported that basic fibroblast growth factor enhances PPARγ2 ligand-induced adipogenesis in rat MSC [46], and FGFR1 is a key regulator of adipogenesis in human [47,48]. In the present study, we also found higher expression of FGFR1 in cells treated with DMIOA compared to non-treated cells showing the involvement of this transcript in chicken adipogenesis.

### The effect of 20(S) hydroxycholesterol on adipogenesis and expression of adipogenic genes

Studies have indicated that specific oxysterols are capable of inhibiting the expression of several adipogenic transcription factors and adipogenesis in mammalian cells [15,16,18,19]. Here, we report for the first time that 20(S) inhibits the adipogenic differentiation of preadipocytes in laying hen.

It has been reported that treatment of murine M2-10B4 MSC with 20(S) completely inhibits troglitazone-induced PPARγ2 expression and adipocyte formation through the activation of the Hh signaling pathway [15]. Moreover, 20(S) induces the expression of notch target genes such as HEY 1/2 and HES1 in M2-10B4 pluripotent mouse stromal cells [39]. Osteogenic oxysterols have also been reported to inhibit adipogenesis and the expression of LPL and FABP4 in murine pluripotent MSC [16]. In this study, treatment of laying hen preadipocytes with 20(S) inhibited the mRNA expression of adipogenic differentiation marker (FABP4) and fatty acid transporter molecule (CD36) (Additional files 5 and 6). However, PPARγ2, LPL, C/EBPβ, C/EBPα and other adipogenic transcription factors were not differentially expressed between DMIOA and DMIOA + 20(S) treated cells. This could be attributed to the fact that these key adipogenic genes are induced during the earlier stages of adipogenic differentiation and hence their mRNA changes were not detected at the later stages, where cells already differentiated. Although 20(S) inhibits PPARγ2 expression and adipogenesis through the activation of the Hh signaling pathway in mouse MSC [15], Hh signaling pathway was not activated by 20(S) treatment in the present study explaining inter-species differences. This suggests that the anti-adipogenic effects of 20(S) may not be mediated through Hh signaling pathway in chicken. Additionally, the network analysis of genes over-expressed in DMIOA treated cells relative to DMIOA + 20(S) showed large proportion of molecules involved in lipid metabolism indicating the potential of 20(S) to reduce excessive fat accumulation (Figure 9). We found over-expression of genes such as INSIG1, STARD4, DHCR7 and 24 and others in cells treated with DMIOA compared with those treated with DMIOA + 20(S). Studies have indicated that INSIG1 is expressed in parallel with FABP4 in differentiating 3 T3-L1 mouse preadipocytes [49], and STARD4 mediates cholesterol transport during cholesterol homeostasis [50]. Genes involved in cell-to-cell signaling networks such as IL1, IL-1R, IL1RL2, CCL20, TNFAIP3, IKB, TNIP2, CSF3 and RIPK2 were also over-expressed in preadipocytes treated with DMIOA + 20(S) compared with those treated with DMIOA (Figure 10). Interleukin 1 (IL-1) plays an important role in bone metabolism through activation of receptor activator of NF-κB signaling pathway in mouse models [51], and IL-1R is an important mediator involved in many cytokine induced immune and inflammatory responses [52]. Additionally, it has been shown that cytokines such as IL-1 and tumour-necrosis factor-alpha (TNFA) inhibit adipogenesis in bone marrow [53]. Hence, over-expression of IL-1, its receptor mRNA (IL-1R) and tumor necrosis alpha induced protein 3 (TNFAIP3) in cells treated with DMIOA +20(S) relative to those treated with DMIOA indicates that 20(S) potentially inhibits chicken adipogenesis through induction of anti-adipogenic cytokines. However, the role/s of cytokines on adipogenesis and adipogenic gene expression in hen preadipocytes need further elucidation.

Genes over-expressed in preadipocytes treated with DMIOA + 20(S) relative to those treated with DMIOA alone include KLF2 and 6, early growth response 1 (EGR1), CD44, and CCK. The Kruppel-like factor (KLF2) has been implicated to reduce adipogenesis by inhibiting PPARγ expression in 3 T3-L1 cells [54]. Similarly, ectopic expression of EGR1 has been reported to inhibit adipocyte differentiation in murine 3 T3-L1 preadipocytes [55]. Consistent with these findings, KLF2 and EGR1 were over-expressed in cells treated with DMIOA + 20(S) relative to cells treated with DMIOA where adipogenesis was markedly reduced. Interestingly, these genes were over-expressed in control cells relative to those treated with DMIOA, indicating that KLF2 and EGR1 could potentially involve in the inhibition of adipogenesis in chicken fat cells.

Studies have shown that increased CD44 expression is accompanied with obesity-induced hepatic steatosis and white adipose tissue-associated inflammation in human and mouse, suggesting CD44 might play a critical role in regulating obesity and associated pathologies [56,57]. On the other hand, because it is a multifunctional cell membrane protein, CD44 can act as a receptor for hyaluronan (HA) and osteopontin [58,59]. In line with this, exogenous HA application increased calcium deposition in pig bone marrow stromal cells [59], and osteopontin expression was enhanced during bone formation in mouse [60], indicating the possible involvement of CD44 in bone development. Considering the potential of 20(S) to induce the expression of genes associated with osteogenesis in M2-10B4 bone marrow stromal cells [15], increased expression of CD44 in cells treated with DMIOA + 20(S) in the present study may explain the pro-oesteogenic property of 20(S) [15] which could negatively modulate key adipogenic regulators by enhancing the expression of CD44. The expression of CCK was also increased in hen preadipocytes treated with DMIOA + 20(S) compared with those treated with DMIOA alone. Cholecystokinin (CCK) is involved in regulating the metabolic rate and is important for lipid absorption and control of body weight in mice placed on a high-fat diet [61], showing its anti-adipogenic effect in vivo. However, the potential of this gene as an anti-adipogenic factor in hen adipocytes needs further investigation.

In summary, treatment of hen preadipocytes with DMIOA highly induced the expression of genes involved in lipid metabolism relative to non-treated cells. On the other hand, preadipocytes treated with a combination of DMIOA and 20(S) inhibited expression of key adipogenic transcripts and adipogenesis as compared with cells treated with DMIOA alone. Moreover, 20(S) increased the expression of many key genes previously reported to enhance osteogenesis which has a reciprocal relationship to adipogenesis.

## Conclusions

This study generated important gene expression data that would enhance our understanding of the biology of adipocytes. The study demonstrated that 20(S) is capable of reducing DMIOA-induced adipogenesis and identified potential adipogenic and anti-adipogenic regulators in hen preadipocytes that require further investigation.

### Supporting data

All supporting data for this study are included as additional files.

## Additional files

Additional file 1:
**List of genes over-expressed (Fold change ≥ 2) in control cells relative to cells treated with an adipogenic cocktail (DMIOA) for 96 hr.**
Additional file 2:
**List of genes over-expressed (Fold change ≥ 2) in cells treated with an adipogenic cocktail (DMIOA) for 96 hr.**
Additional file 3:
**Hierarchical clustering and heat map of all genes differentially expressed between control cells and cells treated with an adipogenic cocktail (DMIOA) for 96 hr.** Numbers 1 and 2 at the top of the heat map indicate the three biological replications representing control cells and cells treated with DMIOA, respectively. The fold change increases from green to red. Additional file 4:
**Hierarchical clustering and heat map of the top 20 genes differentially expressed between control cells and cells treated with an adipogenic cocktail (DMIOA for 96 hr.** Numbers 1 and 2 at the top of the heat map indicate the three biological replications representing control cells and cells treated with DMIOA, respectively. The fold change increases from green to red. Additional file 5:
**List of genes over-expressed (Fold change ≥ 2) in cells treated with an adipogenic cocktail (DMIOA) for 96 hr.**
Additional file 6:
**List of genes over-expressed (Fold change ≥ 2) in cells treated with an adipogenic cocktail (DMIOA) for 96 hr.**
Additional file 7:
**Hierarchical clustering and heat map of all genes differentially expressed between cells treated with an adipogenic cocktail (DMIOA) for 96 hr.** Numbers 1 and 2 at the top of the heat map indicate the three biological replications representing cells treated with DMIOA and DMIOA + 20(S), respectively. The fold change increases from green to red. Additional file 8:
**Hierarchical clustering and heat map of the top 20 genes differentially expressed between cells treated with an adipogenic cocktail (DMIOA) for 96 hr.** Numbers 1 and 2 at the top of the heat map indicate the three biological replications representing cells treated with DMIOA and DMIOA + 20(S), respectively. The fold change increases from green to red.

## Abbreviations

DMIOA: Dexamethasone + 3-isobutyl-1-methylxanthine + insulin + 300 oleic acid
OA: Oleic acid
C/EBPβ: The core enhancer binding protein beta
C/EBPα: The core enhancer binding protein alpha
GSK3β: Glycogen synthase kinase −3- beta
MAPK: Mito gene activated protein kinase
MSC: Mesenchymal stem cell
PKA: Protein kinase A
PKC: Protein kinase C
Hh: Hedgehog
Min: Minutes
Hr: Hour
DMEM: Dulbecco’s Eagle’s Medium
RPM: Revolution per minute
FBS: Fetal bovine serum
GCRMA: Guanine cytosine robust multichip average
qRT-PCR: Quantitative real-time reverse transcriptase polymerase chain reaction
NCBI: National Centre for Biological Information
