# Supplementary figures and images for: Transcriptome analysis of hen preadipocytes treated with an adipogenic cocktail (DMIOA) with or without 20(S)-hydroxylcholesterol
Source: BMC Genomics. 2015 Feb 18;16(1):91. doi: 10.1186/s12864-015-1231-z (PMC4347561; doi:10.1186/s12864-015-1231-z)

## Slide 1
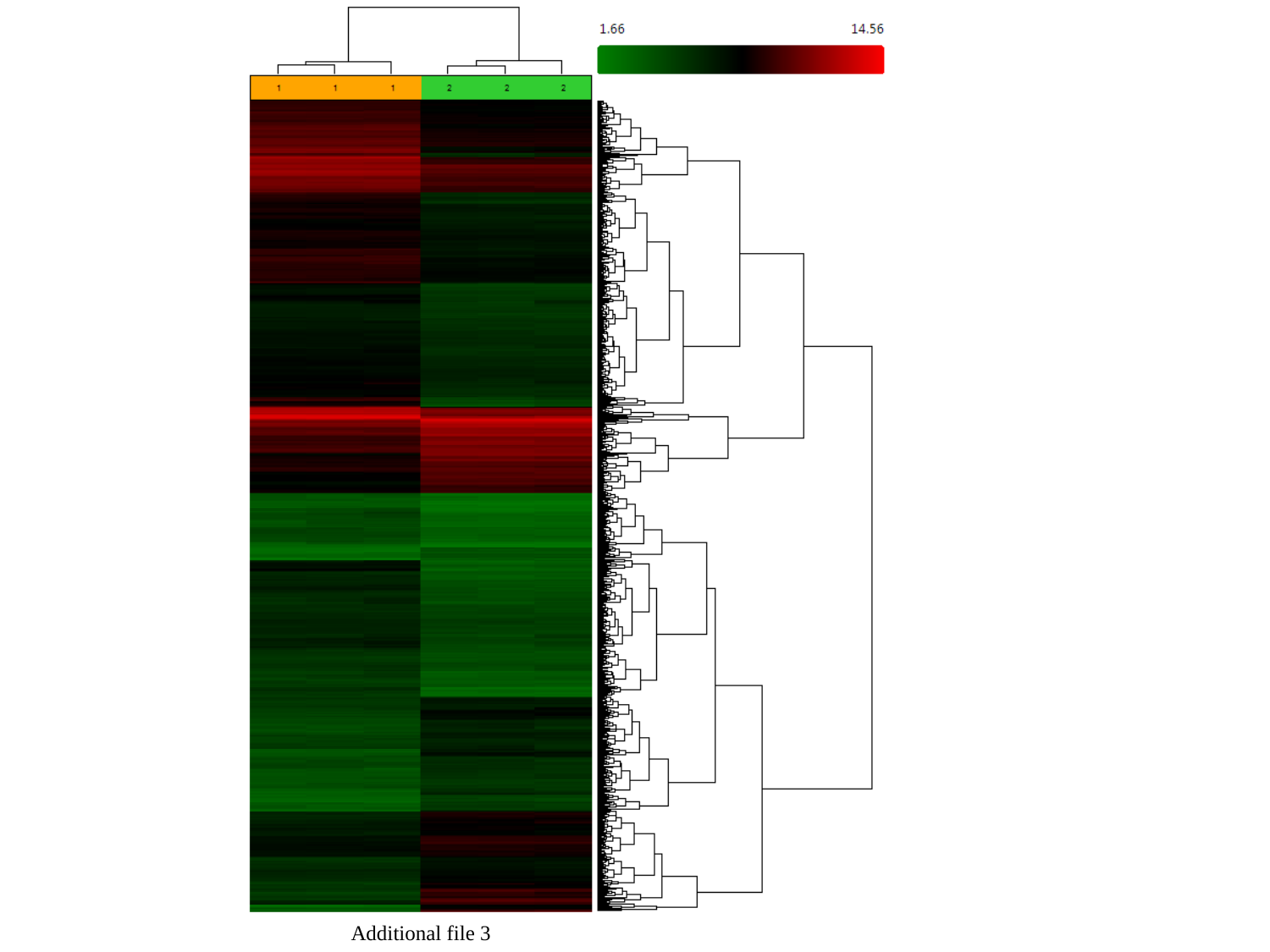

Additional file 3

Supplement: Additional file 3: — Hierarchical clustering and heat map of all genes differentially expressed between control cells and cells treated with an adipogenic cocktail (DMIOA) for 96 hr. Numbers 1 and 2 at the top of the heat map indicate the three biological replications representing control cells and cells treated with DMIOA, respectively. The fold change increases from green to red. [file 12864_2015_1231_MOESM3_ESM.pptx]

## Slide 1
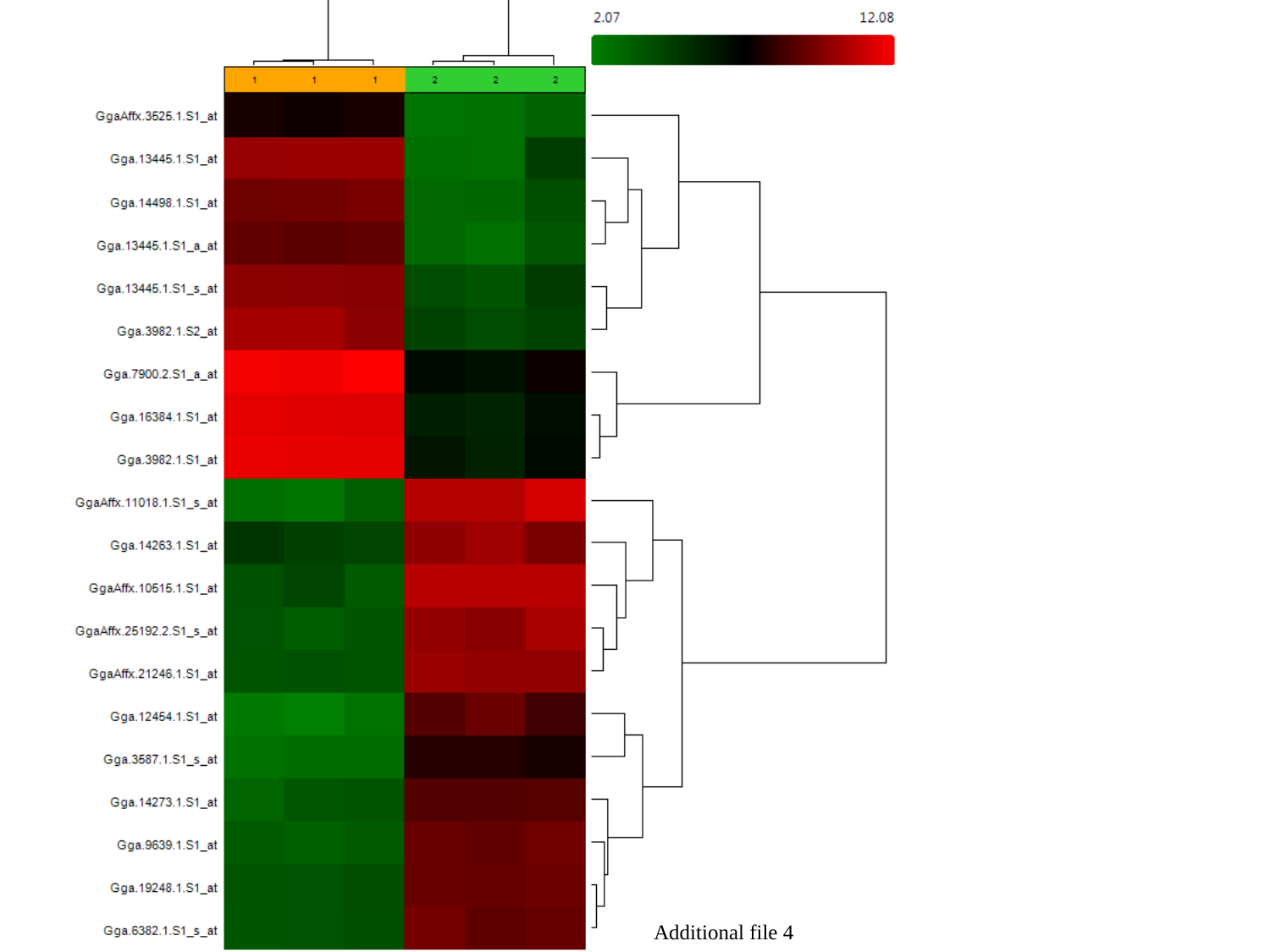

Additional file 4

Supplement: Additional file 4: — Hierarchical clustering and heat map of the top 20 genes differentially expressed between control cells and cells treated with an adipogenic cocktail (DMIOA for 96 hr. Numbers 1 and 2 at the top of the heat map indicate the three biological replications representing control cells and cells treated with DMIOA, respectively. The fold change increases from green to red. [file 12864_2015_1231_MOESM4_ESM.pptx]

## Slide 1
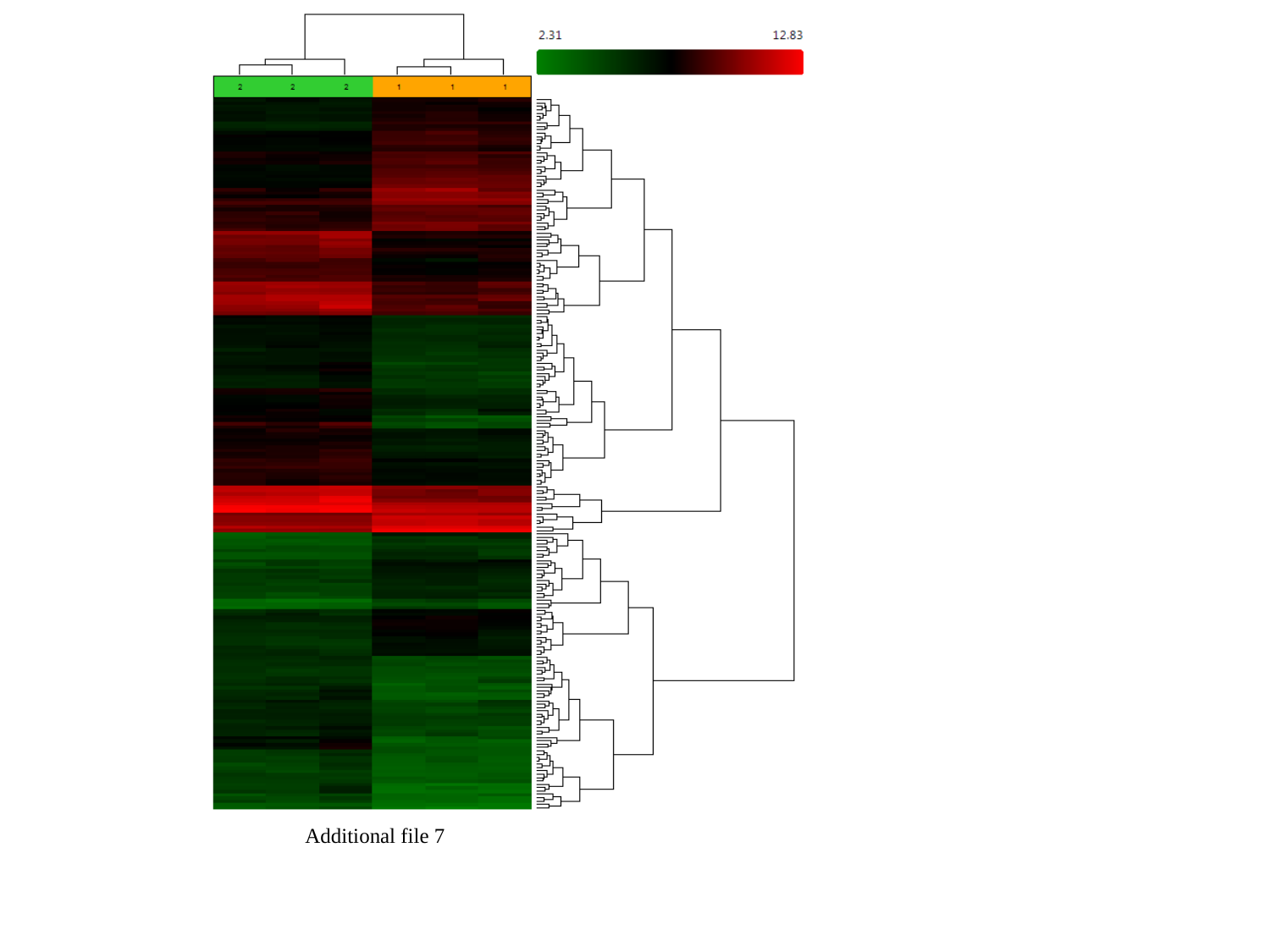

Additional file 7

Supplement: Additional file 7: — Hierarchical clustering and heat map of all genes differentially expressed between cells treated with an adipogenic cocktail (DMIOA) for 96 hr. Numbers 1 and 2 at the top of the heat map indicate the three biological replications representing cells treated with DMIOA and DMIOA + 20(S), respectively. The fold change increases from green to red. [file 12864_2015_1231_MOESM7_ESM.pptx]

## Slide 1
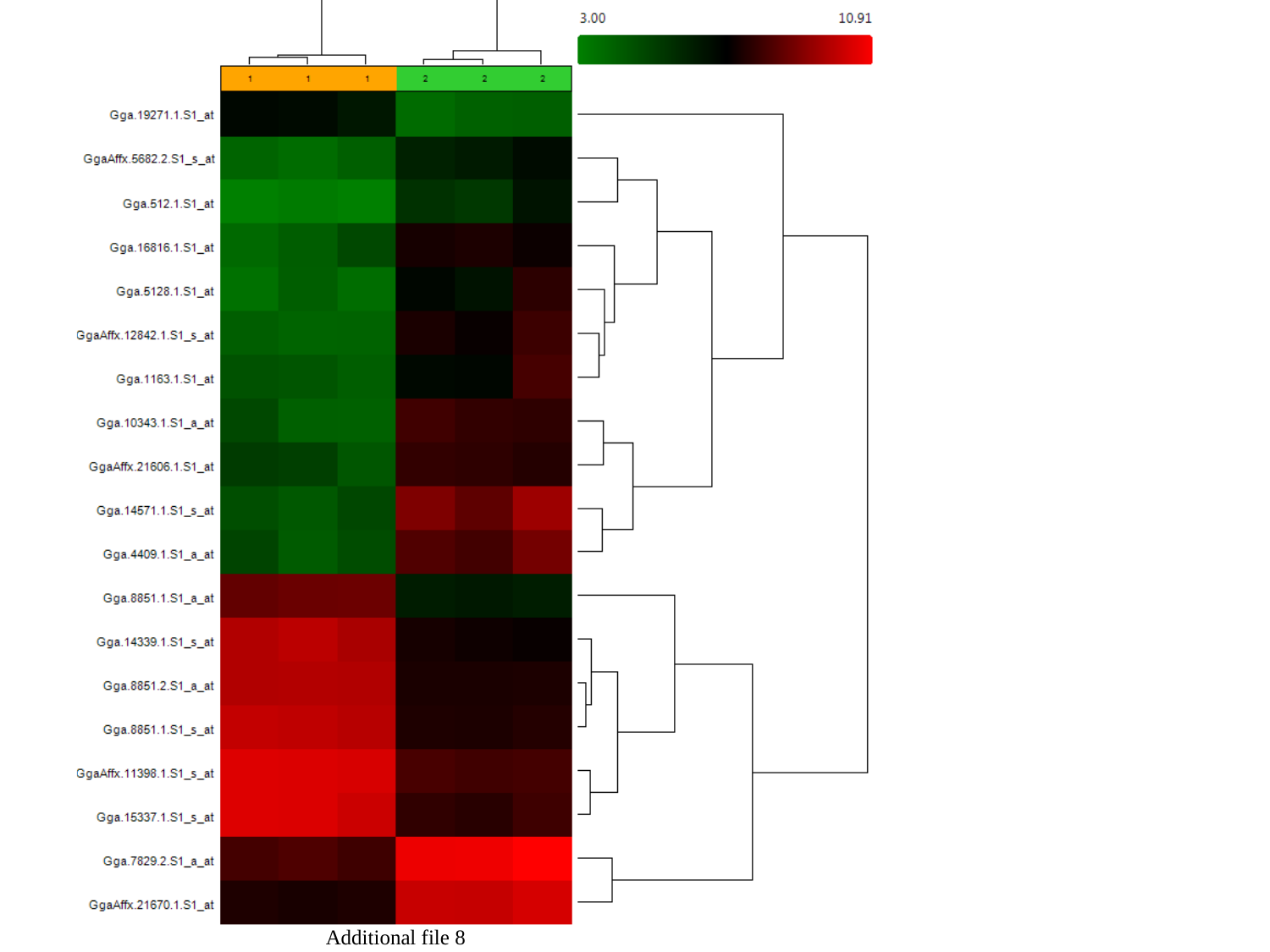

Additional file 8

Supplement: Additional file 8: — Hierarchical clustering and heat map of the top 20 genes differentially expressed between cells treated with an adipogenic cocktail (DMIOA) for 96 hr. Numbers 1 and 2 at the top of the heat map indicate the three biological replications representing cells treated with DMIOA and DMIOA + 20(S), respectively. The fold change increases from green to red. [file 12864_2015_1231_MOESM8_ESM.pptx]
